# The Dynamic Change of Immune Checkpoints and CD14+ Monocytes in Latent Tuberculosis Infection

**DOI:** 10.3390/biomedicines9101479

**Published:** 2021-10-15

**Authors:** Ping-Huai Wang, Ming-Fang Wu, Chi-Yu Hsu, Shu-Yung Lin, Ya-Nan Chang, Ho-Shen Lee, Yu-Feng Wei, Chin-Chung Shu

**Affiliations:** 1 Division of Pulmonology, Department of Internal Medicine, Far Eastern Memorial Hospital, New Taipei City 220, Taiwan; pinghuaiwang@gmail.com; 2 Graduate Institute of Toxicology, College of Medicine, National Taiwan University, Taipei 100, Taiwan; wmf680102@gmail.com; 3 Institute of Statistical Sciences, Academia Sinica, Taipei 100, Taiwan; 4 College of Medicine, National Taiwan University, Taipei 100, Taiwan; thebesthst@gmail.com (C.-Y.H.); simlin0330@gmail.com (S.-Y.L.); 5 Department of Internal Medicine, National Taiwan University Hospital, Taipei 100, Taiwan; 6 Department of Medical Research, National Taiwan University Hospital, Taipei 100, Taiwan; allen6888@gmail.com; 7 Division of Chest Medicine, Department of Internal Medicine, E-Da Hospital, Kaohsiung 824, Taiwan; leehoshn@gmail.com (H.-S.L.); yufeng528@gmail.com (Y.-F.W.); 8 School of Medicine for International Students, College of Medicine, I-Shou University, Kaohsiung 824, Taiwan

**Keywords:** CD14+ monocytes, immune checkpoint, latent tuberculosis infection

## Abstract

Controlling latent tuberculosis infection (LTBI) is important for preventing tuberculosis (TB). However, the immune regulation of LTBI remains uncertain. Immune checkpoints and CD14+ monocytes are pivotal for immune defense but have been scarcely studied in LTBI. We prospectively enrolled participants with LTBI and controls from January 2017 to December 2019. We measured their CD14+ monocytes and the expression of immune checkpoints, including programmed death-1 (PD-1), cytotoxic T-lymphocyte-associated protein 4 (CTLA-4), and T cell immunoglobulin mucin domain-containing-3 (TIM3) on T lymphocytes in peripheral blood mononuclear cells before and after LTBI treatment. A total of 87 subjects were enrolled, including 29 IGRA-negative healthy controls (HC), 58 in the LTBI group (19 without chronic kidney disease (non-CKD), and 39 with end-stage renal disease (ESRD)). All PD-1, CTLA-4, and TIM3 on lymphocytes and monocytes were higher in the LTBI group than that in the HC group. Total CD14+ monocytes were higher and PD-L2+CD14+ over monocytes were lower in patients with LTBI-non-CKD than that in the HC group. After LTBI treatment, CD14+ monocytes, TIM3+ on CD4+ and monocytes, and CTLA-4 on lymphocytes decreased significantly. Multivariable logistic regression indicated that CD14+ monocytes was an independent factor for LTBI-non-CKD from the HC group, whereas PD-L2+CD14+ monocytes and TIM3+ monocytes were significant for LTBI-ESRD from the HC group. In conclusion, LTBI status was associated with increasing CD14+ monocytes plus low PD-L2 expression. By contrast, increased expression of immune checkpoints over all immune cells might be due to *Mycobacterium tuberculosis* related immune exhaustion, which decreased after treatment.

## 1. Introduction

Tuberculosis (TB) is still one of the most common infectious diseases in the world [1]. Prevention of latent TB infection (LTBI) reactivation is an important strategy to reduce TB incidence, in addition to optimal TB treatment [2]. However, the process of *Mycobacterium tuberculosis* (Mtb) changing from LTBI to TB reactivation is not well understood [3]. In particular, why one subject has LTBI, but another does not is still a puzzle even after several decades [4]. Understanding the immune nature of LTBI may help us improve its diagnosis and management.

Immune checkpoints, including programmed death-1 (PD-1), cytotoxic T-lymphocyte-associated protein 4 (CTLA-4) and T cell immunoglobulin mucin domain-containing-3 (TIM3), have been found to be pivotal switches in immunity adjustment for T lymphocytes [5] and are used in routine cancer therapy at present [6,7]. However, many infections, such as viral hepatitis [8] and active tuberculosis [9,10], are reportedly controlled by immune checkpoints. Notably, LTBI with presentation of granuloma is a typical presentation of immune tolerance, and dynamic change in immune checkpoints may be involved in LTBI development, but current research on this possibility is insufficient [11].

On the other hand, innate immunity is the first line of defense after Mtb enters the respiratory system. Classical monocytes in the blood with CD14+ expression are the major sources of alveolar macrophages, and they can provide antigen presentation and help phagocytosis [12]. In addition, the PD-1 ligands 1 and 2 (PD-L1 and 2) may regulate the function of monocytes [13,14] and affect the tolerance of innate immunity to LTBI. However, the above-mentioned immune checkpoints and associated pathways have never been studied in LTBI. We therefore conducted this study to investigate the association between LTBI and the expression of immune checkpoints as well as CD14+ monocytes. In addition, we monitored their dynamic change before and after LTBI treatment to understand the possible causal relationship.

## 2. Materials and Methods

### 2.1. Patient Enrollment

In the present prospective study, we enrolled subjects examined for LTBI from January 2017 to December 2019. Diagnosis of LTBI was defined as positive interferon-gamma release assay (IGRA) using the QuantiFERON-TB Gold In-tube (QFT-GIT) test. This study was conducted at three hospitals in northern Taiwan, including National Taiwan University Hospital (NTUH), its Jin-Shan branch, and Far Eastern Memorial Hospital (FEMH). Approval for the study was granted by the respective Research Ethics Committees (No. 201609062RIND (26 Jan 2017), 201709038RINB (25 Oct 2017), and 108067-F (27 June 2019),). All of the participants provided written informed consent. Exclusion criteria for the study included pregnancy, known infection of human immunodeficiency virus, recent treatment with chemotherapy or corticosteroids, and evidence of active tuberculosis infection.

### 2.2. Study Group and Design

After enrollment, the participants were classified as the LTBI group if their QFT-GIT were positive, whereas those in the control group had negative QFT-GIT results. Participants with end-stage renal disease (ESRD) were part of the LTBI group. ESRD is a well-known risk population for TB reactivation due to its impaired immunity and requires LTBI intervention [15]. Thereafter, participants in LTBI group were classified into two subgroups, namely, the non-CKD subgroup and the ESRD subgroup, according to whether they received dialysis or not in order to check the effect of ESRD. The subjects with LTBI were offered LTBI treatment of weekly high doses of isoniazid and rifapentine for 12 weeks or daily isoniazid for nine months if there was no contraindication and the participants agreed.

### 2.3. Blood Sampling and Cell Isolation

Peripheral blood from the enrolled subjects was sampled into heparin-containing tubes. The timing of sampling in the LTBI group was before and 12 weeks after LTBI treatment, regardless of treatment regimen. Peripheral blood mononuclear cells (PBMC) were immediately isolated with Ficoll-Paque PLUS (GE Healthcare Life Sciences, Sweden) and then suspended in medium containing RPMI-1640 (Life Technologies, Carlsbad, CA, USA), 10% fetal bovine serum (FBS, Biological Industries, Cromwell, Connecticut, USA), and 1% penicillin-streptomycin (Life Technologies, Carlsbad, CA, USA). All cells were resuspended in 10% DMSO and 90% FBS, and were then stored in liquid nitrogen. We defrosted the cells within one week for the scheduled experiments.

### 2.4. Flow Cytometry for PBMCs or Peripheral Blood Lymphocytes

We cultured PBMCs overnight (around 16 h) after the cells were defrosted. The PBMCs were examined directly without any stimulation. Before we stained the PBMCs, we blocked the non-specific bindings of antibody to Fc receptors on monocytes by using Human TruStain FcX™ (Fc Receptor Blocking Solution) (Biolegend, San Diego, CA, US) according to manufacturer’s instruction. The PBMCs were then stained for two panels: (1). CD4, CD8, TIM3, PD-1 and CTLA-4; and (2). CD14, PD-L2, PD-L1, and galactin-9. The staining antibodies were anti-CD4-APC, anti-TIM3-PerCP, anti-CTLA-4- PE-Cy7 (Biolegend, San Diego, CA, USA), anti-CD8-FITC, and anti-PD-1-PE (BD Biosciences, San Diego, CA, USA) in panel 1. Anti-CD14-PerCP, anti-Galectin9-PE (Biolegend, San Diego, CA USA), anti-PD-L1-FITC, and anti-PD-L2-APC antibodies (BD Biosciences, San Diego, CA, USA) were in panel 2. Antibodies were diluted according to the manufacturer’s recommendation and we used 2% FBS in phosphate buffered saline (PBS) as statin buffer.

The data were measured using flow cytometry (FACSVerse, BD Biosciences, USA) with automatic compensation. The PMT voltage and final compensation parameters were demonstrated in Tables S1 and S2 (the Supplementary File S1). Data were gated by unstaining and isotype staining control. We then measured and analyzed the results by BD FACSuite V software (BD, Biosciences, USA). Briefly, in panel 1, we discriminated the lymphocyte and monocyte populations by forward scatter and side scatter. We gated the subgroups of CD4^+^ or CD8^+^ T lymphocytes in the lymphocyte population. The expressions of PD-1, TIM3 and CTLA-4 were then measured in the CD4+ and CD8+ sub-populations and monocyte population. In panel 2, we gated CD14+ cells in the monocyte population and measured the expression of galectin-9, PD-L1, and PD-L2 (Figure S1 in the Supplementary File S1).

### 2.5. Data Collection

Demographics including age, gender, smoking, body mass index, and underlying comorbidities were reviewed by the study nurse. Current smokers were defined as those who had smoked >100 cigarettes, with the last time of smoking within one month prior to the study [16]. In addition, radiographic findings and laboratory data at enrollment were recorded. Chest radiographic findings were labeled as no lesion, lesion not related to TB, or lesion compatible with prior TB [17,18]. If any radiographic lesions were found, sputum mycobacterial culture was performed to exclude the presence of active TB.

### 2.6. Statistical Analysis

Demographics were compared among the control group, LTBI-non-CKD group and LTBI-ESRD group by Mann–Whitney U test for continuous variables and the *chi*-squared test for categorical variables. Wilcoxon pair test was used for comparing data before and after LTBI treatment. Logistic regression was used to analyze factors associated with the LTBI-non-CKD or LTBI-ESRD groups. Multivariable analysis included factors which were statistically significant in univariate analysis. Receiver operating characteristic (ROC) curves were used for examining the performance of the independent factors from logistic regression. The Youden index was applied to set a cut-off value. All statistical analyses were performed in SPSS (Version 19.0, IBM, Chicago, IL, USA) and GraphPad Prism 8.00, GraphPad Software.

## 3. Results

### 3.1. Demographics of the Participants

During the study period, a total of 87 subjects were enrolled in this study. Among them, 29 subjects were in the IGRA-negative health control group (HC), and the other 58 subjects were in the IGRA-positive LTBI group (LTBI-ALL). Of these, 39 were receiving regular hemodialysis (LTBI-ESRD group) and 19 had normal renal function (LTBI-non-CKD group). The demographic data are shown in Table 1. Participants in the LTBI-ESRD group were older than those in the LTBI-non-CKD group (61.5 ± 9.8 vs. 49.1 ± 15.7, *p* = 0.003). Males were more predominant in the LTBI-ESRD group than in the LTBI-non-CKD group (71.8% vs. 47.4%, *p* = 0.019). There were more smokers in LTBI-ALL group (13, 22.4%) than HC group (3, 10.3%), who mostly were subjects with hemodialysis (12, 30.8%). The co-morbidities and radiologic findings on prior TB were not different in these three groups. Hemoglobin level was highest in the HC group, followed by the LTBI-non-CKD and then LTBI-ESRD groups (13.8 ± 1.5, 12.5 ± 2.0, and 11.4 ± 1.5 g/dL, *p* < 0.05 between any two of the three groups). However, white blood cell (WBC) counts were not significantly different.

### 3.2. Immune Checkpoint Expression in LTBI Status

The data on the baseline percentages of immune checkpoint expression on CD4+ and CD8+ in lymphocytes, total monocytes, and CD14+ in monocytes are listed in Table 2. The percentages of CD4 and CD8 cells in lymphocytes were not significantly different among the three groups. The TIM3, PD1 and CTLA-4 expressions on CD4+ and CD8+ in lymphocytes and total monocytes were all significantly higher in the LTBI-non-CKD group than in the HC group, except for the percentage of CTLA-4 expression on monocytes (CTLA-4+monocyte) with a marginally significantly lower value (6.6 ± 5.3 vs. 11.8 ± 10.0, *p* = 0.065). In addition, the percentage of PD-L2 expression on CD14+ (PD-L2+CD14+) monocytes was lower in the LTBI-non-CKD group than in the HC group (50.4 ± 27.2 vs. 69.0 ± 28.3, *p* = 0.025). However, total CD14+ monocytes were higher in the LTBI-non-CKD group than in the HC group (46.7 ± 26.3 vs. 23.4 ± 18.4, *p* = 0.003).

**Figure 1:**
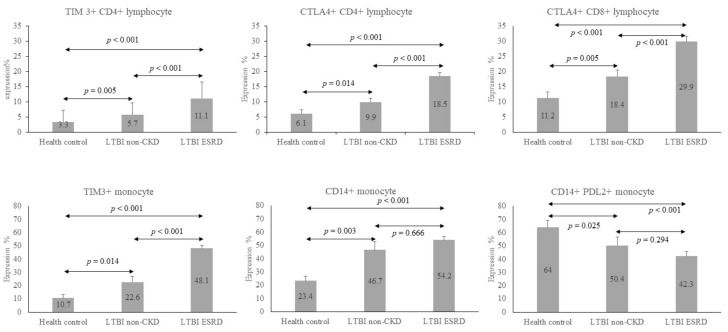
Bar chart to illustrate the expression status of selected immune checkpoints and CD14+ monocytes of health control (HC), LTBI-non-CKD, and LTBI-ESRD. The number in the bars were the mean and the error lines represented mean of standard deviation. ↔ meant comparison of two groups. Only the percentages of TIM3 and CTLA-4 expression on CD4+ cells (TIM3+CD4+ and CTLA-4+CD4+), CTLA-4+CD8+, and TIM3+ monocytes were significantly higher in the LTBI-ESRD group than in the LTBI-non-CKD group. They also were significantly higher in LTBI-non-CKD than HC. CD14+ expression of monocyte was significantly higher in HC than LTBI-non-CKD, but not in LTBI-ESRD. On the contrast, PDL2+ CD14 expression of monocytes were significantly higher in LTBI-non-CKD, compared to HC. The data between various groups were compared by Mann–Whitney U test.

Similar results were found between the HC and LTBI-ESRD groups, except that the percentage of PD1 expression on CD8+ (PD1+CD8+) T cells was not significantly higher in the LTBI-ESRD group than in the HC group (29.9 ± 11.1 vs. 26.8 ± 11.7, *p* = 0.204). However, the percentage of PD-L1 expression on CD14+ (PD-L1+CD14+) monocytes was significantly lower in the LTBI-ESRD group than in the HC group (5.4 ± 3.9 vs. 12.7 ± 9.3, *p* = 0.001). However, the differences between the immune checkpoint expressions of the LTBI-non-CKD and LTBI-ESRD groups were less significant. Only the percentages of TIM3 and CTLA-4 expression on CD4+ cells (TIM3+CD4+ and CTLA-4+CD4+), CTLA-4+CD8+ and TIM3+ monocytes were significantly higher in the LTBI-ESRD group than in the LTBI-non-CKD group (Figure 1).

### 3.3. Immune Checkpoint Expression after LTBI Treatment

**Figure 2:**
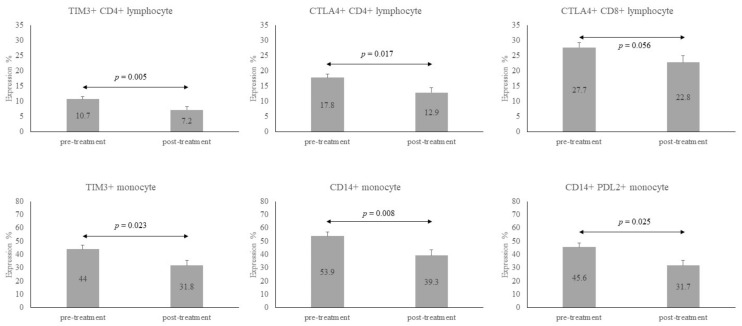
Bar chart to illustrate the dynamic changes of immune checkpoints and CD14+ monocytes before and after treatment of latent tuberculosis infection (LTBI). The number in the bars were the mean and the error lines represented mean of standard deviation. ↔ meant comparison of two groups. All these six subtypes of CD4+, CD8+, and monocytes were significantly decreased after LTBI treatment, except for CTLA4+ CD8+ lymphocyte, which was marginally significant. The data before treatment was compared to those after treatment by Wilcoxon pair test.

Twenty-six subjects (66.7%) in the LTBI-ESRD group and 7 (36.8%) in the LTBI-non-CKD completed LTBI treatment. Their results were pooled, and the individual changes in these immune checkpoint expressions on various immune cells between baseline and 12 weeks of LTBI treatment are listed in Table 3. Thirty subjects received weekly high doses of isoniazid and rifapentine for a total of 12 doses, and the remaining 3 received daily isoniazid for 9 months. The percentage of CD4+ lymphocytes decreased after 12 weeks of LTBI treatment (42.5 ± 11.2 vs. 36.6 ± 15.1, *p* = 0.005). TIM3-CD4 lymphocytes, CTLA-4+CD4+ lymphocytes, TIM3+Monocytes, total CD14+, and PD-L2+CD14+ monocytes were less expressed after 12 weeks of LTBI treatment as compared with the baseline (p = 0.005, 0.017, 0.023, 0.008, and 0.025, respectively). The expression of CTLA-4+CD8+ tended to be lower after treatment, with marginal significance (22.8 ± 12.9 vs. 27.7 ± 11.4, *p* = 0.056) (Figure 2). In summary, TIM3+CD4+, CTLA-4+CD4+, CTLA-4+CD8+, and TIM3+ monocytes, as well as CD14+ monocytes, were higher in the LTBI group than that in the HC group, but they all decreased after 12 weeks of LTBI treatment.

The relationships of the above items among these three groups (HC, LTBI-non-CKD and LTBI-ESRD) and the changes from baseline to 12 weeks are illustrated in Figure 1. Expressions of TIM3+CD4+, CTLA-4+CD4+, TIM3+ monocytes and CTLA-4+CD8+ were lowest in the HC group, higher in the LTBI-non-CKD group, and highest in the LTBI-ESRD group. In contrast to these immune checkpoint expressions, expression of PD-L2+CD14+ monocytes was highest in the HC group and became lower in LTBI groups. All tended to regress after 12 weeks of LTBI treatment.

### 3.4. Predictors for LTBI in Logistic Regression

**Figure 3:**
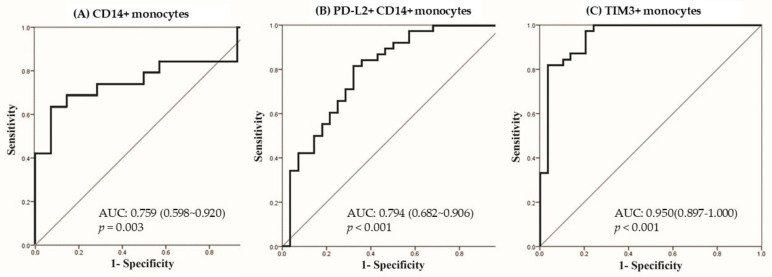
Receiver operating characteristic curves to predict latent tuberculosis infection (LTBI) of (**A**) total CD14+ monocytes among LTBI-non-CKD and healthy controls, (**B**) PDL2+ CD14+ monocytes, and (**C**) TIM3+ monocytes among healthy controls and LTBI-ESRD, using significant factors from multivariable logistic regression. The Youden index was applied to set a cut-off value.

Multi-variable logistic regression analysis using a stepwise method by significant factors in univariate analysis showed that only the total CD14+ monocyte was a significant factor for LTBI-non-CKD in comparison with the HC group (OR: 1.046, 95% CI: 1.016–1.077, per 1% increment, *p* = 0.003). The area under the receiver operating characteristic curve (AUROC) was 0.759 (95% confidence interval (CI): 0.598–0.920, *p* = 0.003), as shown in Figure 3A. The cut-off point of total CD14+ monocytes was 51.85% with sensitivity of 0.632 and specificity of 0.929 by the Youden index. Comparing the LTBI-ESRD and HC groups, PD-L2+CD14+ monocytes (OR: 0.963, 95% CI: 0.928–0.999, per 1% increment, *p* = 0.045) and TIM3+ monocytes (OR: 1.123, 95% CI: 1.063–1.186, per 1% increment, *p* < 0.001) were independent factors in multivariable logistic regression. The AUROCs of PD-L2+CD14+ monocytes (Figure 3B) and TIM3+ monocytes (Figure 3C) were 0.794 (95% CI: 0.682–0.906, *p* < 0.001) and 0.950 (95% CI: 0.897–1.0, *p* < 0.001). The cut-off value for TIM3+ monocytes was 13.66% with sensitivity of 1.0 and specificity of 0.786, and the cut-off value of PD-L2+CD14+ monocytes was 40.58% with sensitivity of 0.605 and specificity of 0.782.

## 4. Discussion

In the present study, TIM3, PD1, and CTLA-4 expressions on CD4+ and CD8+ lymphocytes and monocytes were all significantly higher in the LTBI group than that in the HC group. In addition, PD-L2+CD14+ monocytes were lower but the CD14+ monocytes were higher in the LTBI-non-CKD group when compared with the HC. Among the patients with ESRD, LTBI had similar pattern and was associated with lower PD-L1+CD14+ monocytes additionally. After LTBI treatment, the levels of CD14+ monocytes, TIM3+CD4+, CTLA-4+CD4+, CTLA-4+CD8+, and TIM3+ monocytes decreased significantly. Multivariable logistic regression indicated that CD14+ monocyte was an independent factor for LTBI-non-CKD from the HC group. In comparing the LTBI-ESRD and HC groups, PD-L2+CD14+ and TIM3+ monocytes were independent factors for LTBI.

LTBI is an immune diagnosis with exclusion of active TB status [4,19]. After *M. tuberculosis* (Mtb) bacilli enter the lung environment, macrophages kill the bacilli with a multidisciplinary strategy [3]. Once the host macrophage cannot eradicate the Mtb in case of Mtb related immunity evasion [20], lymphocytes will engage to form granuloma [11,21]. The immune balance between Mtb and the host leads to the LTBI status. However, the role of immune checkpoints for the tolerance in LTBI has been scarcely reported in non-cancer patients before [22,23].

We investigated the immune cells and the expression of immune checkpoints in the participants with LTBI and controls. In the present analysis, increased CD14+ monocytes were the independent factor for LTBI. In fact, CD14+ expression has been reported as a marker of active tuberculosis [14], indicating its defense role against TB. The circulating monocyte could immigrate to infected tissue as macrophages and dendritic cells, which is the important part of innate immunity. Some cytokines derived from monocytes were reported as inflammatory markers of TB disease status, such as osteopontin and interleukin-1 beta (IL-1β) [24,25]. Osteopontin is chemoattractive protein and IL-1β is a product of inflammasome from innate immunity of monocyte or macrophage. Both are increasingly expressed in Mtb infection with activation of monocyte/macrophage, echoing our finding of increasing CD14+ monocytes in LTBI status. However, few reports directly measuring CD14+ monocytes in LTBI can be found in the literature [14]. In the present study, we found that CD14+ monocytes were significantly higher in the LTBI group. In contrary, CD4+ and CD8+ lymphocytes from PBMC were similar between the LTBI and HC groups. Actually, CD14+ monocytes have been found to be primed for phagocytosis, immune responses and migration, as well as antigen presentation [12]. This finding might suggest that CD14+ monocytes would be primed for Mtb defense in the LTBI stage because alveolar macrophages cannot kill Mtb after phagocytosis [20] due to an evasion mechanism in the LTBI host. On the monocytes, PD-L1 and PD-L2 tend to decrease to help increase the immunity of CD14+ monocytes [13]. For clinical practice, if IGRA is a borderline value for LTBI, CD14+ monocytes for LTBI without renal impairment and PD-L2+CD14+ monocytes plus TIM3+ monocytes in LTBI with ESRD could be helpful because negative conversion is not uncommon [26].

On the other hand, PD-1, CTLA-4, and TIM3 were all increased on CD4+, CD8+ lymphocytes and monocytes. Although they are only representatives of immune checkpoints, these increases might be caused to counteract the inflammatory response to the initial stage of Mtb infection [21]. Among the immune checkpoints, TIM3+CD4+, CTLA-4+CD4+, CTLA-4+CD8+, and TIM3+ monocytes were significantly lower with CD14+ monocytes after LTBI treatment, suggesting that some of them might be an evasion mechanism due to Mtb. Furthermore, the increases might also be an exhaustion mechanism for tolerance of chronic Mtb infection [10,27]. Notably, not only the lymphocytes surrounding the granulomas but also those in peripheral blood have over-expressed immune checkpoints, leading to a tolerance of LTBI status. The details of tolerance activation in peripheral blood still requires future investigation.

The immune checkpoint inhibitors such as CTLA4, PD1, and PDL1 inhibitors, are used for many advanced cancer therapies [28]. It can resume the anti-tumor activity and avoid tumor tolerance of immunity [29]. However, increased immunity by immune checkpoint inhibitors might not only cause some autoimmune-like unwanted effects, but also worsen TB host control [3]. There were more and more reports of TB reactivation after immune checkpoint inhibitors [30]. Although the pathogenesis remains unclear, the broken hemostasis between Mtb bacilli and immunity by immune checkpoint inhibitors might lead to excessive but ineffective protection to Mtb [31]. Jayaraman and his colleagues reported that the effect of Tim3–Galectin 9 on antimicrobial immunity was a balance between its negative effects on T cells and its enhancement of bactericidal pathways in macrophage and dendritic cells [32]. However, the effect of immune checkpoints and the inhibitors in LTBI needs long-term observation and future intervention study to investigate.

The present study has some strengths and limitations. With regard to the strengths, this study is the first investigation conducted on immune checkpoints in LTBI status. The results provide some perspectives on this unexplored area. Second, the study design was not only cross-sectional but also contained a cohort of patients before and after LTBI treatment, providing deeper understanding of the causal relationship.

Regarding the study limitations, we used cryopreserved cells for the experiment, which might create a lesser response than the fresh cells, but the profiles were comparable [33,34]. To decrease the bias, we performed all the samples in the same cryopreservation protocol and gated them carefully using a surface marker. Notably, many literatures studying the immune cell function using frozen PBMCs have been reported [35,36]. Therefore, our study might show the right profiles of the immune pattern in LTBI although the effect might be underestimated. Second, the case number was small but the differences between the LTBI and HC groups were statistically significant. Third, the case enrollment was conducted in a medical center, so some selection bias might exist. Fourth, we did not enroll CKD patients without LTBI for comparison, the LTBI prediction in CKD cannot be completely analyzed. Fifth, this study examined associations only, and the pathogenesis will require further bench experiments and in-vivo animal study for validation. Sixth, there was no data by plasma inflammatory markers, real time PCR, and Western blot to validate the results in the present study and it needs to be performed in a future study. Last, the study was conducted in Taiwan, so whether the results can be generalized to other ethnicities or regions should be validated.

In conclusion, LTBI status was associated with immunity change with major increases in CD14+ monocytes with low PD-L2+ expression, indicating innate immunity might be activated to defend TB systemically. By contrast, PD-1, CTLA-4, and TIM3 on lymphocytes were higher in LTBI subjects than the HC group, possibly due to an evasion of immune exhaustion in Mtb infection. The dynamic change of host immunity and Mtb evasion are very important to help further LTBI management and study.

## Figures and Tables

**Table 1:** Demographic data of the enrolled participants.

	Healthy Controls (*n* = 29)	LTBI-ALL (*n* = 58)	LTBI-ALL	
			LTBI-Non-CKD (*n* = 19)	LTBI-ESRD (*n* = 39)
Age, year	57.7 ± 15.0 *	57.4 ± 13.3	49.1 ± 15.7 ^¶^	61.5 ± 9.8
Male sex	17 (58.6) *	37(63.8)	9 (47.4) ^¶^	28 (71.8)
Smoking	3 (10.3) ^#§^	13(22.4)	1 (5.3)	12 (30.8)
BMI	23.6 ± 3.8	23.7 ± 3.6	23.7 ± 4.1	23.7 ± 3.4
Underlying disease				
ESRD	0	39(67.2)	0	39(100)
Malignancy	0	1(1.7)	1 (5.3)	0
Cirrhosis of liver	0	0	0	0
Diabetes mellitus	6 (20.7)	8(13.8)	1 (5.3)	7 (17.9)
Radiographic findings				
No lesion	26 (89.7)	55(94.8)	18 (94.7)	37 (92.5)
Lesion, not related to TB	3 (10.3)	3(5.2)	1 (5.3)	3 (7.7)
Lesion, prior TB	0	0	0	0
Hemoglobin, g/dL	13.8 ± 1.5 *^#§^	11.8 ± 1.7	12.5 ± 2.0 ^¶^	11.4 ± 1.5
Blood leukocyte count,/μL	6920 ± 1398	7050 ± 2332	7560 ± 2607	6801 ± 2178

Abbreviations: BMI, body mass index; CKD, chronic kidney disease; ESRD, end stage renal disease; LTBI, latent tuberculosis infection; TB, tuberculosis Data are presented as number (%) or mean ± standard deviation. * Statistical significance between healthy controls and LTBI-non-CKD group. ^#^ Statistical significance between healthy controls and LTBI-ESRD group. ^¶^ Statistical significance between LTBI-non-CKD and LTBI-ESRD group. ^§^ Statistical significance between healthy controls and LTBI-ALL group.

**Table 2:** Immune checkpoints and immune cell proportions according to status of latent tuberculosis infection and renal function. The data are shown in percentage.

	Healthy Controls (*n* = 29)	LTBI-non-CKD (*n* = 19)	LTBI-ESRD (*n* = 39)
CD4+ cells in lymphocytes	43.0 ± 12.9	41.9 ± 13.7	43.0 ± 11.0
TIM3 on CD4+	3.3 ± 3.9 *^#^	5.7 ± 4.0 ^¶^	11.1 ± 5.5
PD-1 on CD4+	17.3 ± 9.4 *^#^	22.1 ± 11.6	25.6 ± 10.1
CTLA-4 on CD4+	6.1 ± 6.8 *^#^	9.9 ± 5.6 ^¶^	18.5 ± 7.6
CD8+ cells in lymphocytes	22.1 ± 8.3	23.1 ± 11.9	22.3 ± 9.1
TIM3 on CD8+	8.5 ± 8.6 *^#^	12.6 ± 7.0	16.0 ± 7.6
PD-1 on CD8+	26.8 ± 11.7 *	33.1 ± 9.0	29.9 ± 11.1
CTLA-4 on CD8+	11.2 ± 10.7 *^#^	18.4 ± 9.4 ^¶^	29.9 ± 10.6
TIM3 in monocytes	10.7 ± 13.6 *^#^	22.6 ± 18.9 ^¶^	48.1 ± 15.0
PD-1 on monocytes	10.4 ± 10.5 *^#^	16.7 ± 10.9	18.8 ± 8.8
CLTA-4 on monocytes	11.8 ± 10.0 ^#^	6.6 ± 5.3	6.6 ± 5.7
CD14+ on monocytes	23.4 ± 18.4 *^#^	46.7 ± 26.3	54.2 ± 16.8
PD-L1 on CD14+ cells	12.7 ± 9.3 ^#^	12.5 ± 21.6	5.4 ± 3.9
PD-L2 on CD14+ cells	69.0 ± 28.3 *^#^	50.4 ± 27.2	42.3 ± 20.0
Galectin-9 on CD14+ cells	16.2 ± 14.8	19.0 ± 29.4	9.5 ± 7.0

Abbreviations: CKD, chronic kidney disease; CTLA-4, cytotoxic T-lymphocyte-associated protein 4; ESRD, end stage renal disease; LTBI, latent tuberculosis infection; PD-1, programmed death-1, TIM3, T cell immunoglobulin mucin domain-containing-3. Data are presented as mean ± standard deviation. * Statistical significance between healthy controls and LTBI-non-CKD group. ^#^ Statistical significance between healthy controls and LTBI-ESRD group. ^¶^ Statistical significance between LTBI-non-CKD and LTBI-ESRD groups.

**Table 3:** Changes in immune checkpoints and immune cell proportion according to treatment status of latent tuberculosis infection (*n* = 33 pairs). The data are shown in percentage.

	Before LTBI Treatment	After 12 Weeks of LTBI Treatment	*p* Value
CD4+ cells in lymphocytes	42.5 ± 11.2	36.6 ± 15.1	0.005
TIM3 on CD4+	10.7 ± 5.6	7.2 ± 5.8	0.005
PD-1 on CD4+	26.3 ± 11.8	24.1 ± 9.7	0.114
CTLA-4 on CD4+	17.8 ± 7.4	12.9 ± 8.9	0.017
CD8+ cells in lymphocytes	23.5 ± 9.4	20.9 ± 11.4	0.120
TIM3 on CD8+	16.0 ± 7.7	13.7 ± 8.9	0.138
PD-1 on CD8+	31.1 ± 12.9	29.7 ± 11.4	0.265
CTLA-4 on CD8+	27.7 ± 11.4	22.8 ± 12.9	0.056
TIM3 in monocytes	44.0 ± 19.7	31.8 ± 23.1	0.023
PD-1 on monocytes	16.3 ± 8.7	16.5 ± 10.7	0.952
CLTA-4 on monocytes	6.6 ± 4.8	5.6 ± 5.6	0.448
CD14+ on monocytes	53.9 ± 21.0	39.3 ± 23.3	0.008
PD-L1 on CD14+ cells	8.4 ± 14.6	8.2 ± 17.4	0.809
PD-L2 on CD14+ cells	45.6 ± 21.9	31.7 ± 22.3	0.025
Galectin-9 on CD14+ cells	12.3 ± 17.8	9.3 ± 16.8	0.519

Abbreviations: CTLA-4, cytotoxic T-lymphocyte-associated protein 4; LTBI, latent tuberculosis infection; PD-1, programmed death-1, TIM3, T cell immunoglobulin mucin domain-containing-3. Data are presented as mean ± standard deviation.

## Data Availability

The datasets used and/or analyzed during the current study are available from the corresponding author on reasonable request.

## Supplementary Materials

The following are available online at https://www.mdpi.com/article/10.3390/biomedicines9101479/s1, Figure S1: The gating methods for flow cytometry, Table S1: The PMT voltage in the working panel of flow cytometry, Table S2: The compensation values between each two fluorochromes.
