# Supplementary material for: The Dynamic Change of Immune Checkpoints and CD14+ Monocytes in Latent Tuberculosis Infection
Source: Biomedicines. 2021 Oct 15;9(10):1479. doi: 10.3390/biomedicines9101479 (PMC8533229; doi:10.3390/biomedicines9101479)

Table S1. The PMT voltage in the working panel of flow cytometry.

	Voltage
FSC	227
SSC	334
FITC	306
PE	348
PerCP	416
PE-Cy7	522
APC	499

Table S2. The compensation values between each two fluorochromes.

PANEL 1

-% Fluorochrome / Fluorochrome	FITC	PE	PerCP	PE-Cy7	APC
FITC	NA	1.15	0.00	0.14	0.00
PE	30.00	NA	0.00	0.50	0.00
PerCP	3.24	13.00	NA	0.13	0.20
PE-Cy7	0.21	1.70	67.58	NA	0.22
APC	0.00	0.01	20.56	0.02	NA

PANEL 2

-% Fluorochrome / Fluorochrome	FITC	PE	PerCP	PE-Cy7	APC
FITC	NA	1.15	0.00	0.14	0.00
PE	27.50	NA	0.00	0.5	0.00
PerCP	0.24	12.22	NA	0.13	0.20
PE-Cy7	0.21	1.7	67.58	NA	0.22
APC	0.00	0.01	20.56	0.02	NA

Figure S1. The gating methods for flow cytometry. (A) We discriminated the lymphocyte population (red circle) and monocyte population (purple circle) by forward scatter and side scatter. (B) In panel 1, We gated the (C) monocytes and subgroups of (C) CD4+ or (C) CD8+ T cells in the lymphocyte population. The expressions of PD-1, TIM3 and CTLA-4 were then measured in the CD4+ and CD8+ sub-populations and monocyte population. (F) In the panel 2, we gated (G) CD14+ cells in the monocyte population and measured the expression of galectin-9, PD-L1, and PD-L2.

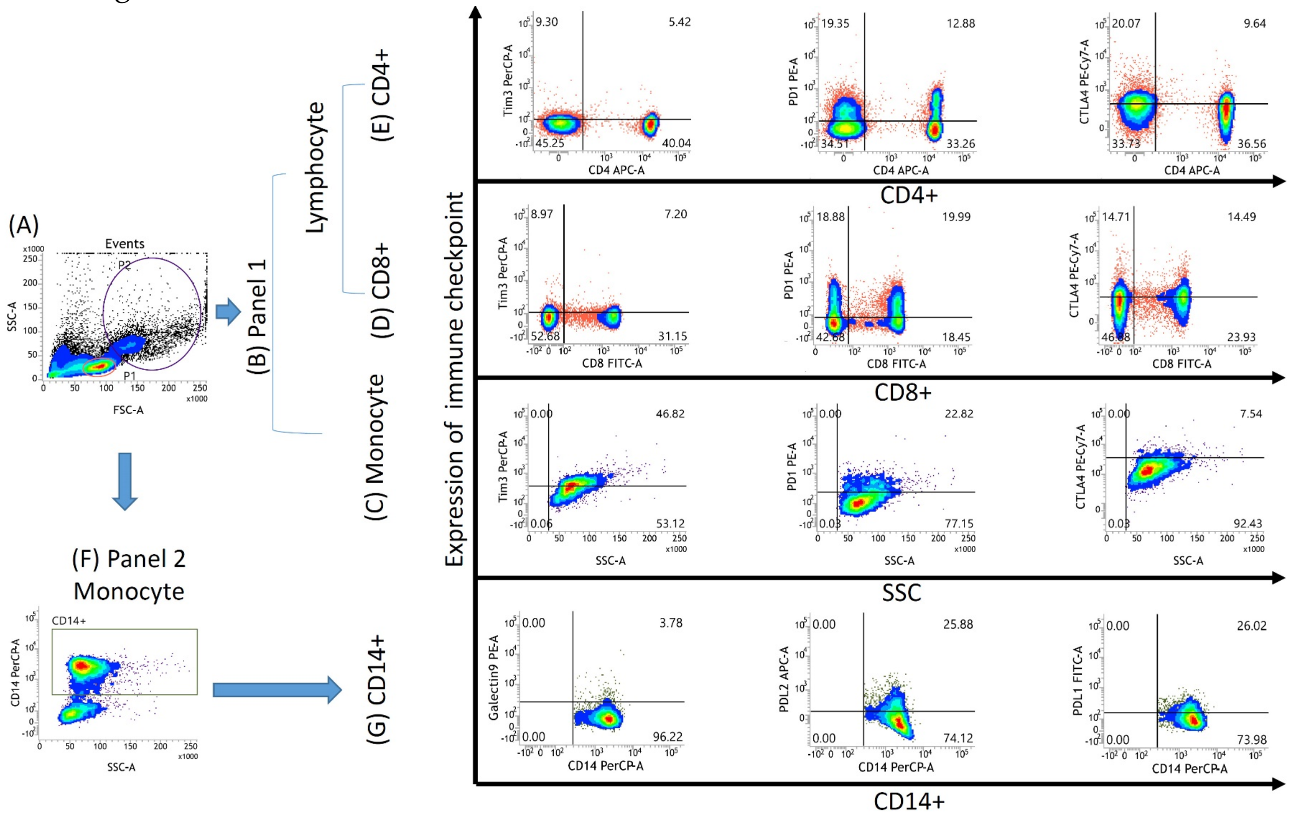

Supplement: Supplementary file 1 [file biomedicines-09-01479-s001.zip › biomedicines-1407685-supplementary.pdf]
